# Clinical Characteristics and Outcomes of Hospitalized Older Patients with Distinct Risk Profiles for Functional Decline: A Prospective Cohort Study

**DOI:** 10.1371/journal.pone.0029621

**Published:** 2012-01-04

**Authors:** Bianca M. Buurman, Jita G. Hoogerduijn, Elisabeth A. van Gemert, Rob J. de Haan, Marieke J. Schuurmans, Sophia E. de Rooij

**Affiliations:** 1 Department of Internal Medicine and Geriatrics, Academic Medical Center, Amsterdam, The Netherlands; 2 Research Group Care for the Chronically Ill, Faculty of Health Care, Hogeschool Utrecht, University of Applied Sciences Utrecht, Utrecht, The Netherlands; 3 Clinical Research Unit, Academic Medical Center, Amsterdam, The Netherlands; 4 Department of Nursing Science, University Medical Center, Utrecht, The Netherlands; Marienhospital Herne - University of Bochum, Germany

## Abstract

**Background:**

The aim of this research was to study the clinical characteristics and mortality and disability outcomes of patients who present distinct risk profiles for functional decline at admission.

**Methods:**

Multicenter, prospective cohort study conducted between 2006 and 2009 in three hospitals in the Netherlands in consecutive patients of ≥65 years, acutely admitted and hospitalized for at least 48 hours. Nineteen geriatric conditions were assessed at hospital admission, and mortality and functional decline were assessed until twelve months after admission. Patients were divided into risk categories for functional decline (low, intermediate or high risk) according to the Identification of Seniors at Risk-Hospitalized Patients.

**Results:**

A total of 639 patients were included, with a mean age of 78 years. Overall, 27%, 33% and 40% of the patients were at low, intermediate or high risk, respectively, for functional decline. Low-risk patients had fewer geriatric conditions (mean 2.2 [standard deviation [SD] 1.3]) compared with those at intermediate (mean 3.8 [SD 2.1]) or high risk (mean 5.1 [SD 1.8]) (p<0.001). Twelve months after admission, 39% of the low-risk group had an adverse outcome, compared with 50% in the intermediate risk group and 69% in the high risk group (p<0.001).

**Conclusion:**

By using a simple risk assessment instrument at hospital admission, patients at low, intermediate or high risk for functional decline could be identified, with distinct clinical characteristics and outcomes. This approach should be tested in clinical practice and research and might help appropriately tailor patient care.

## Introduction

Functional decline, defined as a loss of activities of daily living (ADL), is experienced by 30 to 60% of hospitalized older patients [1], [2]. In acutely hospitalized patients, functional decline often precedes hospital admission [3], and hospitalization itself further increases the risk of worsening ADL disabilities [4]. Patients with functional decline are also at risk for other adverse health outcomes, such as institutionalization and death [5].

Preventing functional decline during and after hospitalization is therefore an increasingly important health-care focus in older hospital patients [6], [7]. Not all patients are at equal risk of developing functional decline because decline is dependent on (among other factors) patients' premorbid status, including geriatric conditions present at admission [1], [8]. The aggregate number of geriatric conditions present at hospital admission determines a patient's individual risk for functional deterioration [1], [9].

In studies focusing on assessing the risk of functional decline, the study population is often crudely dichotomized into a low-risk and a high-risk group [5]. Both the International Classification of Functioning (ICF) and expert opinion suggest the need for patient care and research to adopt a more tailored approach, in which different subgroups or categories of older patients are identified.[7]–[10]. The added value of such an approach is that it might help clinicians define subtle treatment goals at an early stage (for instance, at hospital admission), discuss preferred and expected hospital care outcomes with their patients and it might enhance clinical decision making. Although some studies have attempted to develop an approach using more than two subgroups of patients [11], [12], the clinical characteristics and outcomes of these patients groups have not been described and studied thoroughly [13].

The objectives of this multicenter, prospective, observational study were therefore to investigate 1) differences in the clinical characteristics of patients at low, intermediate or high risk for functional decline, 2) the different functional trajectories from baseline to one year after discharge in the risk groups and 3) the association between risk categories and mortality and functional decline at three and twelve months after hospital admission.

## Methods

### Design and setting

This multicenter prospective cohort study, the DEFENCE study (Develop strategies Enabling Frail Elderly New Complications to Evade) was conducted between April 1, 2006 and April 1, 2008 in three hospitals in The Netherlands: the Academic Medical Center (AMC) in Amsterdam, the University Medical Centre Utrecht (UMCU) in Utrecht and the Spaarne Hospital (SH) in Hoofddorp. The AMC (1,024 beds) and UMCU (1,042 beds) are tertiary university teaching hospitals. The SH (455 beds) is a regional teaching hospital.

In total, five wards in the AMC, three wards in the UMCU and three wards in the SH participated in this study. The staff on the general medical wards consisted of residents, physicians and registered nurses who did not specialize in geriatric medicine or geriatric nursing. A geriatric consultation team consisting of at least one clinical nurse specialist and one geriatrician was available in all hospitals.

The study was approved by the Medical Ethics Committee of the AMC. Local approval was given by the UMCU and SH.

### Patients

The study enrolled all consecutive patients aged 65 years and older who were acutely admitted to one of the three participating hospitals' medical wards and hospitalized for at least 48 hours. Patients were excluded if 1) they or their relatives did not give informed consent; 2) they were too ill to participate, as determined by their attending medical doctor; 3) they came from another ward in or outside the hospital; 4) they were transferred to the Intensive Care Unit of the Coronary Care Unit or another ward in or outside the hospital within 48 hours after admission; or 5) they were unable to speak or understand Dutch. Enrollment had to take place within 48 hours after admission, and written informed consent was obtained before inclusion.

### Data collection

A research nurse visited the participating wards every weekday seeking eligible patients for the study. After obtaining informed consent from the patient or, in case of cognitive impairment, from the primary caregiver, the patient received a risk assessment, followed by a systematic geriatric assessment on four domains of functioning (somatic, psychological, functional and social) performed by the research nurse. The primary caregiver was also interviewed. The patient assessment had to be completed within 48 hours after admission.

### Risk assessment for functional decline

The Identification of Seniors at Risk–Hospitalized Patients (ISAR-HP) was applied to determine which patients were at low, intermediate or high risk for functional decline. The ISAR-HP is based on the original ISAR for the Emergency Department (ED) [14]. The ISAR has been validated to detect a broad range of adverse outcomes after Emergency Department discharge and has been shown to be a clinimetrically sound screening instrument [14]–[16]. The original ISAR was tested on its predictive accuracy in acutely hospitalized older medical patients, but did not show good discriminative values in this population [17]. Therefore, a new prediction model was developed in an independent population and externally validated to assess the risk of functional decline three months after hospital admission in older hospitalized patients. The complete procedure is described in another article [18] . Briefly, in the development study (n = 492) potential predictors associated with functional decline were identified using univariate logistic regression. Items of the original ISAR screening instrument [14], of the IADL index of Lawton and Brody [19], of the Short Nutritional Assessment Questionnaire [20] and other predictors known from the literature were analyzed as individual predictors. Next, a multivariate logistic regression was conducted (backward procedure, accepting P-values≤0.05). The four best models were compared and validated in a bootstrap procedure (1000 samples drawn randomly with replacement) using the AUC with 95% CI to determine the discriminative value. The AUC of the best model was 0.71 (95% CI 0.66–0.76) and the Hosmer Lemeshow test showed a p-value of 0.95, indicating a good fitting model. The validation cohort consisted of a retrospective analysis of a cohort of 484 patients acutely admitted to general medicine ward; the AUC of the prediction model in the validation cohort was 0.68 (95% CI 0.63–0.73).

The screening instrument was named ISAR-HP (with permission of the original author) and consists of four variables 1) the need for assistance with instrumental activities of daily living (IADL) two weeks prior to hospital admission, 2) eight years or fewer of formal education, 3) the inability to travel alone two weeks prior to hospital admission and 4) the use of a walking device. The first three items scored one point each, and the last item scored two points. Patients were at risk for functional decline if the ISAR-HP was 2 points or more. Definition of risk categories applied in this article: low risk if patients scored 0 or 1 point on the ISAR-HP, at intermediate risk if they scored 2 or 3 points and at high risk if patients had a score of 4 or 5.

### Systematic geriatric assessment

At admission, patients' baseline and clinical characteristics were assessed with a comprehensive geriatric assessment (CGA). Table S1 shows the measurement tools, score ranges and cut-off scores used during this assessment. The CGA started with the eleven-item Minimal Mental State Examination (MMSE) [21] to assess the presence and degree of global cognitive impairment. Patients with a MMSE score ≥21 were interviewed; patients with a MMSE score of 16–20 were also interviewed, but their answers concerning baseline characteristics and ADL performances were cross-checked with their caregiver. In case of a disagreement, the caregiver's answer was included. Data for patients with an MMSE score ≤15 were obtained from their primary caregiver. This latter group was not screened for pain, depression or perceived health status, as the instruments we used have not been validated with cognitively impaired patients.

After administering the comprehensive geriatric assessment, the research nurse reported her findings to the geriatrician. The geriatrician also visited each patient within 48 hours and paid special attention to diagnosing potential psychiatric problems. The patient was screened for delirium using the confusion assessment method (CAM) [22].

After discharge, a geriatrician reviewed the discharge letter to determine the medical diagnoses presented at admission, new diagnoses developed during the patient's hospital stay, comorbidities and medication. Charlson comorbidity index scores were derived from this information [23], indicating the number and severity of comorbidities. Charlson comorbidity index scores range from 0 to 31, with a higher score indicating an increased number of severe comorbidities. International Classification of Diseases-9 diagnostic criteria were used to score these diagnoses.

### Follow-up and definition of outcomes

Three and twelve months after admission, a research nurse from each center phoned the patient and/or primary caregiver to assess the patient's current ADL functioning. ADL status was collected from the same person (patient or informal caregiver) from whom the baseline information was obtained. Functional decline was defined as a loss of at least one point on the original Katz ADL index score [24] three or twelve months after admission, compared with the premorbid Katz ADL index score two weeks prior to hospital admission.

The mortality rate at three months and twelve months after admission was based on information from the Municipal Data Registry.

Functional trajectories were defined as the course of functioning from admission up to one year after discharge and were constructed using mortality and functional decline data at each time point. Patients who were still alive at three and twelve months and did not demonstrate decreased ADL functioning remained at their baseline level of function.

### Statistical analysis

Baseline characteristics and outcomes were summarized using descriptive statistics. To determine the differences in the prevalence of geriatric conditions and outcomes among patients at low, intermediate and high risk for functional decline, dichotomous variables and categorical data were tested with a chi-squared test, and continuous variables were tested using ANOVA. For some geriatric conditions there were missing values.

**Figure 1:**
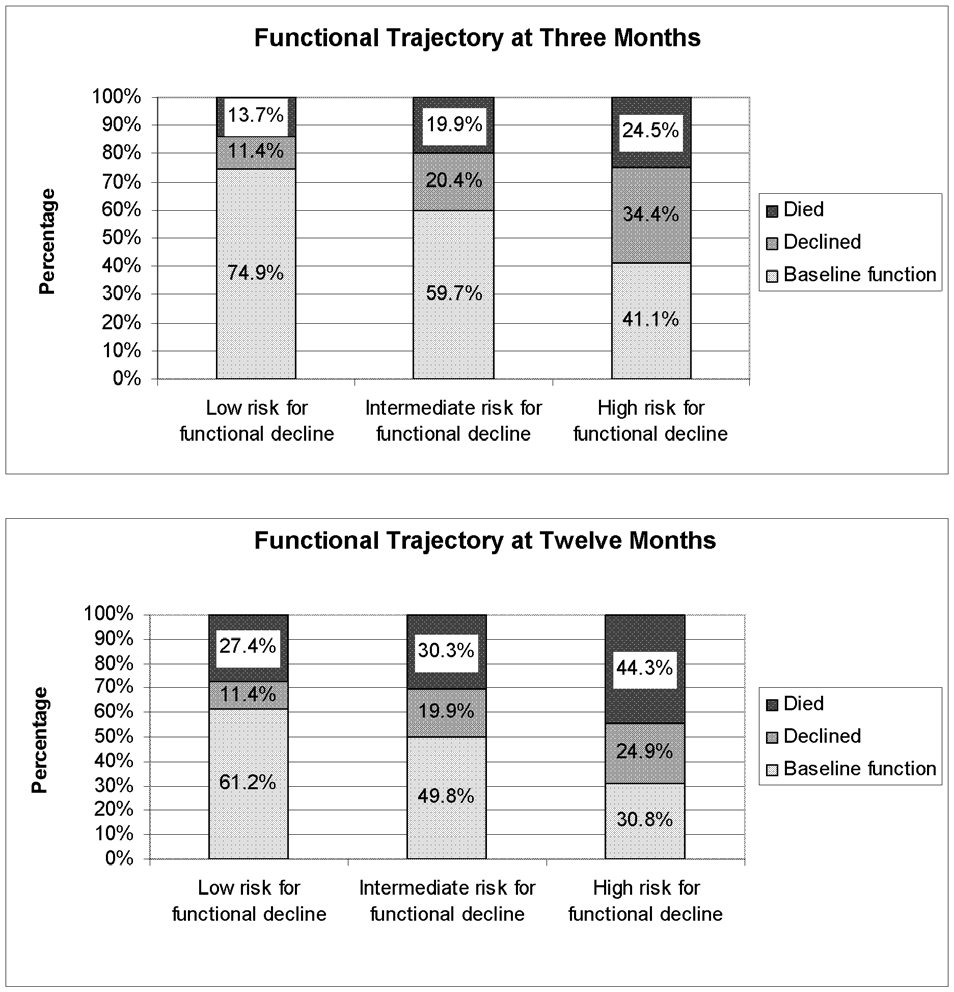
Functional trajectories for patients at low, intermediate or high risk for functional decline three and twelve months after admission. “Baseline function” refers to the level of premorbid functioning on the Katz ADL index score two weeks prior to hospital admission. A decline in function was defined as a loss of at least one point at three or twelve months on the six-item Katz ADL index compared with premorbid functioning.

To gain more insight in the functional trajectories until one year after admission in relation to the ISAR-HP two strategies were followed. One strategy was to calculate the individual responses to the ISAR-HP questions, and to compute the mean number of baseline and follow up scores on the Katz ADL index and the mean number of ADL functions that were lost between baseline and follow up. To establish functional trajectories including mortality at three and twelve months, the number of patients who had died and who demonstrated functional decline in each risk group was calculated. Patients who improved in activities of daily living were added to the group that remained at baseline functional levels. This was set out in figure 1.

To determine the relationship between risk category and mortality and functional decline at three and twelve months, regression analyses were performed. For mortality, Cox regression analyses were performed. Crude and adjusted (for age, sex and Charlson comorbidity index) models were calculated. For functional decline, logistic regression analyses were conducted and crude and adjusted models were computed, adjusting for the same factors. Patients in the low-risk group were used as a reference category.

## Results

There were 1,031 consecutive patients eligible for participation in this study, 639 (62%) of whom were included after informed consent. Reasons for exclusion were refusal to participate (n = 222), insufficient Dutch language capabilities (n = 86), transfer from another ward (n = 36), transfer to Intensive Care Unit or Coronary Care Unit within 48 hours (n = 28) and terminal illness (n = 20). Compared with included patients, excluded patients were significantly younger (75 years vs. 78 years, p<0.001) and died more frequently within one year (48% vs. 35%, p<0.001).

### Baseline characteristics of the three risk groups

Table 1 presents the baseline characteristics of the complete study population. The mean age was 78 years; 72% lived independently before hospital admission and approximately half the patients lived alone. The most common reason for admission was infection (41%). ISAR-HP scores showed that 27%, 33% and 40% of the patients were at low, intermediate or high risk for functional decline, respectively. There was a significant relationship between higher risk levels and older age, female sex, fewer years of education/lower social status, living alone, and care dependency.

**Table 1:** Baseline characteristics of acutely hospitalized older patients in three risk categories for physical functional decline.

	Patients n = 639	Low risk(n = 175)	Intermediate risk(n = 211)	High risk(n = 253)	p-value
Age in years	78.2 (7.8)	73.8 (6.4)	77.4 (7.1)	82.0 (7.5)	<0.001
Male (%)	46.2	60.0	46.9	36.0	<0.001
Education in years	9.9 (3.9)	11.4 (3.8)	10.2 (3.9)	8.6 (3.6)	<0.001
Caucasian (%)	92.8	95.4	91.9	91.7	0.35
**Social status (%)**					<0.001
Living alone	47.9	37.1	46.7	56.3	
**Living arrangement (%)**					<0.001
Independent	72.4	93.7	78.6	52.6	
Senior residence	10.3	4.6	9.0	15.4	
Supported living community	10.3	0.6	6.7	20.2	
Nursing home/intermediate care	7.0	1.1	5.8	11.8	
**Diagnosis at admission (%)**					0.76
Infectious disease	40.9	42.9	45.5	35.9	
Digestive system disease	22.8	23.8	21.8	22.9	
Malignancy	6.2	8.3	4.5	6.1	
Cardiovascular disease	4.3	4.8	2.7	5.3	
Water and electrolyte disturbance	10.5	9.5	8.2	13.0	
Other	15.4	10.7	17.3	16.8	
**Charlson comorbidity index** *	3.5 (2.3)	3.9 (2.7)	3.8 (2.4)	3.5 (2.2)	0.27
**Length of hospital stay in days (median [range])**	7 (2–100)	5 (2–100)	7 (2–77)	8 (2–80)	0.01

Mean (SD) are given for continuous variables.

*Range 0–31; a higher score indicates more or more severe comorbidities.

### Clinical characteristics

Table 2 shows the clinical characteristics of patients at low, intermediate or high risk for functional decline. Patients at high risk for functional decline had more geriatric conditions (mean 5.1 [SD 1.8]) than those at low risk (mean 2.2 [SD 1.3]) or intermediate risk (mean 3.8 [SD 2.1]) for decline (p<0.001). In the high-risk group, patients frequently presented geriatric syndromes, such as fall risk, incontinence, premorbid cognitive impairment and delirium. As expected, there was also a substantial caregiver burden in the high-risk group.

**Table 2:** Clinical characteristics of acutely hospitalized older patients in the three risk categories for physical functional decline.

	Low risk n = 175% (n/total number of observations)	Intermediate risk n = 211% (n/total number of observations)	High risk n = 253% (n/total number of observations)	p-value
**Somatic domain**				
Polypharmacy	46.6 (81/174)	64.8 (136/210)	66.3 (167/252)	<0.001
Malnutrition	45.2 (76/168)	50.5 (105/208)	54.6 (136/249)	0.17
Obesity	8.9 (15/168)	13.8 (26/188)	12.7 (27/213)	0.33
Pain*	42.3 (58/137)	44.5 (77/173)	42.8 (74/173)	0.91
Fall risk	4.2 (7/165)	27.9 (57/204)	30.0 (72/240)	<0.001
Presence of a pressure ulcer	0.0 (0/141)	3.6 (7/196)	4.1 (10/245)	0.06
Indwelling urinary catheter	7.6 (13/172)	20.0 (42/210)	37.3 (94/252)	<0.001
Incontinence	14.5 (24/165)	23.8 (49/206)	24.3 (60/247)	0.04
Constipation	20.3 (35/172)	14.9 (31/208)	22.0 (55/250)	0.15
**Psychological domain**				
Premorbid cognitive impairment	7.4 (9/121)	24.7 (43/174)	42.1 (91/216)	<0.001
Cognitive impairment at time of admission	10.9 (19/175)	34.6 (73/211)	64.8 (164/253)	<0.001
Depressive symptoms*	18.2 (25/137)	20.3 (35/172)	24.7 (42/170)	0.36
Prevalent delirium	2.3 (4/175)	19.2 (40/208)	29.7 (71/239)	<0.001
**Functional domain**				
Premorbid ADL impairment	13.1 (23/175)	50.2 (106/211)	77.3 (194/251)	<0.001
Vision impairment	9.5 (16/169)	20.7 (41/198)	30.5 (75/246)	<0.001
Hearing impairment	13.0 (21/161)	18.1 (35/193)	23.3 (55/236)	0.04
Low health status score*	31.1 (42/135)	38.0 (65/171)	44.0 (74/168)	0.07
**Social domain**				
High perceived caregiver burden	26.3 (31/118)	41.7 (70/168)	50.2 (111/221)	<0.001
**Total number of geriatric conditions** † **(mean (SD))**	2.2 (1.3)	3.8 (2.1)	5.1 (1.8)	<0.001

*Not assessed in patients with severe cognitive impairment, defined as an MMSE score ≤15 at admission.

† not including pain, depressive symptoms and low health status score, as those were most frequently not measured in high risk patients. Only cognitive impairment at admission was included in the total number of geriatric conditions.

ADL = activities of daily living, IADL = instrumental activities of daily living.

We could not demonstrate clear differences between the subgroups with regard to malnutrition, obesity, pain, constipation or depressive symptoms.

### Functional trajectories at three and twelve months

The mean number of baseline impairments on the modified Katz ADL index differed significantly between the three risk groups (0.1, 1.2, 2.4, p<0.001, Table 3). In the low risk group only 13% experienced one or more dependencies in ADL, whereas in the high risk group this was 77%, with 11% demonstrating complete dependence. The mean decline experienced until one year after discharge was also significantly different. Outcomes in terms of mortality and functional decline three and twelve months after hospital admission differed significantly between the groups (Figure 1). After three months, 25% of the low-risk group had a poor outcome (mortality or functional decline), compared with 40% and 59% in the intermediate- and high-risk groups, respectively (p<0.001). At twelve months, these rates were 39%, 50% and 69% for the low-, intermediate- and high-risk group, respectively (p<0.001). Only 30% of the patients in the high-risk group remained at their baseline level of functioning at twelve months. Although the high-risk patients had the most premorbid impairments in ADL, they also deteriorated the most at three and twelve months.

**Table 3:** Response to the ISAR-HP questions, baseline impairments and functional outcomes at three and twelve months in the three risk groups for functional decline.

	Low risk (n = 175)	Intermediate risk (n = 211)	High risk (n = 253)
**Response to ISAR-HP questions**			
1. Needed more help in IADL (% yes)	16.6	32.5	47.6
2. Eight years or fewer of formal education (% yes)	20.8	37.8	58.7
3. Needed help with travelling (% yes)	5.7	48.6	91.3
4. Use of a walking device (% yes)	0.0	56.4	100.0
**Baseline functional characteristics**			
Katz ADL index* (mean/SD)	0.14 (0.39)	1.16 (1.64)	2.36 (2.10)
**No of baseline impairments on Katz ADL index (%)**			
0	86.9	49.8	22.7
1	12.6	23.7	24.7
2	0.0	10.0	11.2
3	0.6	5.2	8.8
4	0.0	3.8	10.8
5	0.0	3.8	12.0
6	0.0	3.8	10.0
**Functional outcome at three months**			
Katz ADL index* (mean/SD)	0.36 (0.76)	1.32 (1.87)	3.05 (2.10)
Functional decline† (mean/SD)	0.20 (0.77)	0.34 (1.47)	0.83 (1.83)
**Functional outcome at twelve months**			
Katz ADL index* (mean/SD)	0.41 (0.73)	1.40 (1.94)	2.77 (2.13)
Functional decline† (mean/SD)	0.24 (0.70)	0.51 (1.85)	0.68 (1.88)

ISAR-HP = identification of seniors at risk-hospitalized patients, IADL = instrumental activities of daily living, ADL = activities of daily living, SD = standard deviation.

*Katz ADL index; range of scores between 0–6, with a higher score indicating more dependence.

† Functional decline was measured with the Katz ADL index and the outcome at three or twelve months was compared to premorbid functioning two weeks prior to hospital admission. These data were only available for those patients still alive at follow up.

### Risk profiles in relation to mortality and functional decline

Tables 4 and 5 show that in both the crude and adjusted models, being at high risk for functional decline was significantly associated with mortality and poor functional health at both time points. Among patients at intermediate risk, the only significant association was found for functional decline at three and twelve months. However, when adjusting for age, sex and level of comorbidity, we could not demonstrate an association between moderate risk and functional decline one year after discharge.

**Table 4:** Cox regression models for three- and twelve-month mortality in relation to risk categories.

Risk category	Three-month mortality Unadjusted HR (95% CI)	Three-month mortality Adjusted* HR (95% CI)	Twelve-month mortality Unadjusted HR (95% CI)	Twelve-month mortality Adjusted* HR (95% CI)
Low risk	Ref	Ref	Ref	Ref
Intermediate risk	1.49 (0.90–2.45)	1.43 (0.85–2.42)	1.15 (0.79–1.67)	1.10 (0.75–1.62)
High risk	1.82 (1.13–2.91)	1.71 (1.01–2.90)	1.81 (1.29–2.54)	1.62 (1.11–2.35)

HR = hazard ratio; CI = confidence interval.

*Adjusted for age, sex and Charlson comorbidity index.

**Table 5:** Logistic regression models for functional decline at three and twelve months in relation to risk categories.

Risk category	Functional decline at three months Unadjusted OR (95% CI)	Functional decline at three months Adjusted* OR (95% CI)	Functional decline at twelve months Unadjusted OR (95% CI)	Functional decline at twelve months Adjusted* OR (95% CI)
Low risk	Ref	Ref	Ref	Ref
Intermediate risk	2.19 (1.21–3.95)	2.07 (1.11–3.89)	2.07 (1.13–3.80)	1.60 (0.81–3.14)
High risk	5.31 (3.04–9.27)	4.48 (2.41–8.35)	4.29 (2.38–7.75)	3.22 (1.63–6.36)

OR = odds ratio; CI = confidence interval.

*Adjusted for age, sex and Charlson comorbidity index.

## Discussion

This multicenter study showed that by applying a simple risk assessment instrument at admission, three subgroups of older patients with distinct clinical characteristics and outcomes could be identified. Twenty-seven percent of the patients were at low risk for functional decline, 33% were at intermediate risk and 40% were at high risk for developing new disabilities. Patients at high risk for further functional decline presented with the highest number of geriatric conditions. High-risk patients were also at the highest risk for poor outcomes in terms of mortality and deterioration in ADL functioning and their mean overall decline in functioning was significantly greater.

The low-risk group, as expected, presented with the fewest geriatric conditions and ADL impairments at admission but still had an average of two geriatric conditions besides the acute and chronic diseases leading to hospital admission. The number of geriatric conditions and premorbid ADL impairments gradually increased in the intermediate- and high-risk groups. The findings on the differences between the subgroups are consistent with other studies that used a more detailed risk classification for functional decline or frailty [9], [13].

The geriatric conditions most often present in the high-risk group (cognitive impairment, delirium, premorbid ADL impairment, urine incontinence and fall risk) reflect the patients' frailty [25], [26] and are known risk factors for future functional decline [1], [8], [26], [27] . The high-risk group presented with the most baseline impairments and the greatest deterioration of ADLs both in percentage and the mean number of decline over the follow-up period. Lost functions are difficult to recover, and new disabilities or impairment reported at discharge that are still present at one month of follow-up are especially difficult to rehabilitate [27]. Patients discharged with new or additional disabilities also have the highest probability of dying in the year after admission [27]. The severity of the acute illness leading to admission is an important risk factor for mortality [28], [29]. This risk factor might explain the still relatively high mortality rates of 27% and 30% in the low- and intermediate-risk groups, respectively, up to one year after admission.

Compared with the low-risk group, the intermediate group showed an increased risk for functional decline at three months, but this increased risk disappeared at one year. A clear association between the high-risk group and mortality and functional decline was demonstrated at both time points. Only one-third of this group maintained baseline function one year after admission. This finding could indicate that the intermediate group has more potential for further rehabilitation after admission compared with the high-risk group, which might be too frail. Research has demonstrated that once patients begin to decline, they are more prone to further decline, even if they have regained their initial level of functioning [30], [31]. More interestingly, one large study on functional decline at the end of life clearly demonstrated that functional trajectories for patients with both organ failure and frailty in the last year of life demonstrated an almost continuous decline in ADL functioning, starting with already many baseline impairments, whereas in patients with end-stage cancer, this decline only starts in the last two or three months of life and these patients predominantly have a good level of ADL functioning [32]. In our study this might also be visible; in the low risk group, many patients died, but did not have much premorbid dependencies. These patients were more frequently cancer patients, whereas in the high risk group, many baseline impairments were present, and these patients demonstrated most decline in the year after hospital admission.

An important question is whether risk status can identify the patients most likely to benefit from multidisciplinary intervention by a geriatric consultation team. Results of a meta analysis of inpatient geriatric rehabilitation argued that subgroup evidence in favor of providing geriatric rehabilitation during and after hospital admission is warranted [33] and that more tailored approaches to patient selection still need to be tested. A recent randomized clinical trial (RCT) focusing on disease management in older heart failure patients divided participants into three risk groups and found that there was a difference in intervention benefits, in terms of both outcomes and costs, in favor of the intermediate-risk group [34]. The authors argued that the low-risk group was too healthy and that the high-risk group too ill to profit from the intervention.

Further research should focus on testing this risk-based approach in acutely hospitalized older patients. This research could be implemented in two ways. The first is an impact study, testing the clinical usefulness of the approach by determining whether the risk assessment outcomes influence decision making and goal setting in both physicians and patients [35]. The second study that could be performed is an RCT using the three risk groups as a basis for goal setting and intervention. The ICF rehabilitation model could inform goals for the low-, intermediate- and high-risk groups [10]. The ICF rehabilitation model identifies several different health strategies, which can be used to determine rehabilitation outcomes. The three health strategies that might be relevant in relation to this study are the preventive health strategy, in which the main purpose is to prevent health conditions and remain functioning. The second strategy is aimed at rehabilitation in which the primary goal should be to restore functioning and the third strategy is supportive care direct towards maintaining quality of life and preservation of autonomy. These strategies might be relevant for the low, intermediate and high risk group, respectively.

Some limitations need to be addressed. First, in our study, we made a predefined selection with one risk assessment instrument, the ISAR-HP. Our main purpose was to demonstrate that a risk assessment instrument can be helpful to detect low-, intermediate- and high-risk patients. Although our study is a multicenter study, using the ISAR-HP for this purpose in other settings might produce different arising from differences in the case mix of patients, leading to a different distribution of the outcome and predictive factors [35]. We clearly demonstrated that this risk-based approach revealed differences in baseline (clinical) characteristics and health outcomes, further enhancing the validity of this screening instrument.

Second, functional decline was operationalized as a one-point decline at follow-up functioning compared with premorbid functioning. For further analyses, we dichotomized the outcome as present or absent. Although this approach is used in most studies of functional decline in hospitalized older patients [2], it leads to a loss of information about the ADL functioning level after hospitalization.

Third, the inclusion percentage was 62%. This expected but still low inclusion rate is a common problem in studies of acutely hospitalized older patients, and most trials conducted in this population demonstrated equal or lower participation rates [36]–[38]. We did conduct a small non-respondent analysis in which we demonstrated that the patients that were excluded were often younger and died more frequently after discharge. Presumably, these patients more frequently had end stage diseases or were very frail older patients. It would have strengthened the validity of study results, if we would have collected more baseline information on these patients.

### Conclusion

In conclusion, by using an easily applied risk assessment instrument at hospital admission, three patients groups (low, intermediate and high risk for functional decline) with distinct clinical characteristics could be distinguished. This approach might contribute to better defining of treatment goals at hospital admission, earlier initiation of appropriate (preventive) interventions and better communication with patients and caregivers about the preferred outcomes of admission. The application of this approach and the effectiveness of risk-based clinical interventions should further be tested in clinical practice and randomized clinical trials.

## Supporting Information

Table S1 **Content of the systematic comprehensive geriatric assessment.**
(DOC)
